# Multi-Material Topology Optimization of Flexure Hinges Using Element Stacking Method

**DOI:** 10.3390/mi13070993

**Published:** 2022-06-24

**Authors:** Min Liu, Yifeng Li, Jinqing Zhan

**Affiliations:** 1 School of Mechatronics & Vehicle Engineering, East China Jiaotong University, Nanchang 330013, China; lmin2016@foxmail.com (M.L.); liyifeng313@163.com (Y.L.); 2 State Key Laboratory of Performance Monitoring and Protecting of Rail Transit Infrastructure, East China Jiaotong University, Nanchang 330013, China; 3 Key Laboratory of Conveyance Equipment, East China Jiaotong University, Ministry of Education, Nanchang 330013, China

**Keywords:** multi-material flexure hinge, topology optimization, compliant mechanism, element stacking method

## Abstract

Traditional flexure hinges are designed by using a single material, and their performance is inadequate, compared to the ideal hinge. This paper presents a topology-optimization design method for multi-material flexure hinges based on the element stacking method. A topology optimization model for multi-material flexure hinges is constructed to find the optimal distribution of various materials, where the objective function is to maximize the compliance in the rotational direction, whilst minimizing the compliance in the axial direction. In order to ensure the rotation precision of the hinge, the position constraint of the rotation center is proposed. The gradient information of objective and constraint functions is derived by the adjoint method, and the method of moving asymptotes (MMA) is used to update the design variable. Several numerical examples are performed to verify the effectiveness of the proposed method, and the results show that the multi-material flexure hinge has a higher rotation ratio than the single-material flexure hinge.

## 1. Introduction

A compliant mechanism is a kind of structure that can transmit force and movement through elastic deformation, and it is usually processed from a piece of plate material by wire cutting. The jointless compliant mechanism with monolithic construction has the advantages of no friction and wear, no need for lubrication, no assembly, and an ultrahigh accuracy and response speed. Due to the advantages of the compliant mechanism, it has been widely applied in the field of precision engineering, such as micro/nano operations [1,2], the precision positioning stage [3], atomic force microscopes [4], biomedical science [5], MEMS [6], and optical-fiber alignment [7]. According to whether there is a flexure hinge in the mechanism, it can be classified into distributed and flexure-based compliant mechanisms. However, it is difficult to obtain the closed form model of the distributed compliant mechanisms. In engineering applications, the flexure-based compliant mechanism is more widely used because the deformations only occur in the flexure hinges. Since the flexure hinge has a great influence on the performance of the flexure-based compliant mechanisms, it has been extensively studied by researchers [8,9,10,11,12,13,14,15,16,17,18,19,20].

To realize the rotation function, the simplest configuration of the flexure hinge is usually designed as a notch type. Among them, circular [8] and leaf [9] notched flexure hinges are the most basic and common flexure hinges. The circular hinge has the higher rotational precision, and the leaf hinge has the larger range of motion. With the expansion of the application of the compliant mechanism, these two flexure hinges are not enough to meet the requirements. Subsequently, various other notched flexure hinges were presented and studied, such as conic-section (parabolic [10], hyperbolic [10], elliptical [11] and elliptical-arc [12]); corner-filleted [13]; V-shaped [14]; power-function-shaped [15]; polynomial [16]; exponent-sine-shaped; [17] and corrugated [18] flexure hinges. For achieving higher performances, the researchers turned the aforementioned notched flexure hinges combination into hybrid flexure hinges [19,20]. Recently, this design idea has become popular, such as the configuration that employs circular and elliptical profiles, hyperbolic and corner-filleted segments, and so on. However, these flexure hinges are designed based on the designers’ inspiration and experience, and this trial-and-error method does not have general guiding significance.

In order to find the optimal shape or configuration of the flexure hinge, researchers have tried various methods such as the shape optimization approach [21,22] and topology optimization method [23]. The topology optimization method has huge advantages in the configuration design of flexure hinges due to its large design space. This method can find the optimal distribution of materials in a design domain to obtain the best designs when subjected to a given set of boundary conditions, and it does not require the designer to have extensive experience, thereby avoiding design blindness. Zhu et al. [24,25] first designed rotational and translational flexure hinges based on the density-based topology optimization method, and then adopted the level-set method to optimize the shape and topology of a flexure hinge. Liu et al. [26] presented a quasi-V-shaped flexure hinge by fitting the contour curve of the topology result of the flexure hinge, and derived its compliance, precision, and the maximum stress equations. Afterwards, Liu also carried out a lot of research on the topology optimization of a flexure hinge. For example, he used the stress-based topology optimization to design a flexure hinge and reduce its stress level [27], presented a multi-notched flexure hinge on the basis of the right-circular flexure hinge [28], and proposed the topology optimization method of the distributed flexure hinges with desired performance [29,30]. Qiu et al. [31] designed a multicavity flexure hinge using 3D-continuum topology optimization and derived its dimensionless-stiffness empirical equations based on a finite element analysis. Pinskier et al. [32] investigated the topology optimization of stiffness-constrained flexure hinges for maximizing precision and range of motion.

However, in the research mentioned above, many single-material flexure hinges are presented and applied, but multi-material flexure hinges are rare. For example, to improve the dynamic positioning accuracy of the compliant mechanism, Chen et al. [33] proposed a damped leaf flexure hinge, that is, a layer of damping material is attached to the surface of a leaf flexure hinge made of traditional metal materials. By including both a stiff and flexible material phase in the design of a flexure hinge, the vibration of the flexure hinge is obviously suppressed. However, Chen et al.’s design was limited by their intuitive understanding of how a flexure hinge should look. For the single-material flexure hinges, it is difficult to achieve the comprehensive requirements of static and dynamic performances at the same time. If the flexure hinge is designed by combining stiff and flexible materials—that is, different materials are distributed in different parts, according to the performance requirements of the flexure hinge—this can further increase the rotation range of the flexure hinge and ensure the accuracy and dynamic performance. So, how to optimally distribute various materials to improve the comprehensive performance of the flexure hinge? This paper takes a more systematic design approach based on topology optimization to design multi-material flexure hinges.

Multi-material design can realize specific purposes that may be difficult to be attained by single-material structures, or that may find it challenging to appropriately distribute different materials to obtain the optimal performance. Sigmund and Torquato [34] first presented multi-phase topology optimization based on the homogenization method to design three-phase composites with an extremal thermal coefficient. Recently, the multi-material topology optimization has been applied to the design of compliant mechanisms. Chu et al. [35] presented a level set-based topology optimization method to design the multi-material compliant mechanisms with stress constraints. Rostami and Marzbanrad [36] proposed and analyzed the identification of optimal topologies for multi-material compliant mechanisms based on the regularized projected-gradient approach. The SIMP (solid isotropic material with penalization) material interpolation scheme is used to parameterize the continuum design domain. Alonso et al. [37] presented the multiple Sequential Element Rejection and Admission (SERA) method to design multi-material compliant mechanisms.

Many approaches have been developed for multi-material topology optimization and may be classified as density methods (homogenization/SIMP based) [38,39], phase field methods [40], and level set methods [41]. However, the level set method and the phase field method have several major limitations, such as slow convergence, oscillation, local minima, and dependency of the initial guesses; moreover, the mixture of isotropic and anisotropic materials is not supported by the SIMP method. To overcome these challenges, Yoon et al. [42] proposed an element stacking method for multi-material topology optimization, which and Li and Kim [43] improved. This method can transform the formulations of standard multi-material topology optimization into a series of equivalent single-material topology optimization formulations, in order to overcome the various limitations that are inherent to conventional approaches [44]. In this work, the element stacking method is used to perform the topology optimization design of multi-material flexure hinges.

In this paper, Section 2 introduces the element stacking method. In Section 3, the optimization model for the topology optimization of a multi-material flexure hinge is proposed, and the sensitivity analysis is given. Section 4 discusses the numerical implementation aspects. The conclusions are given in Section 5.

## 2. Element Stacking Method

**Figure 1:**
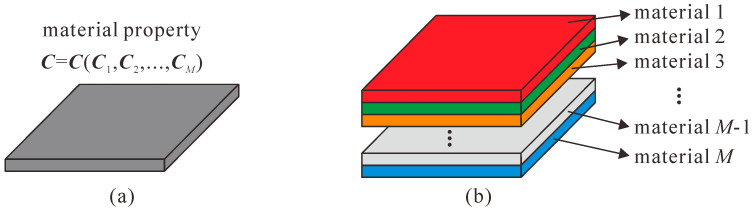
Multi-material models of multi-material topology optimization. (**a**) The conventional method using a single finite element; (**b**) the element stacking method selecting only one element from multiple elements.

In conventional multi-material topology optimization (such as the well-known SIMP method), the design domain is discretized by pixels, with only one finite element per pixel. Therefore, the properties of the pixel are equal to the properties of the corresponding finite element, as shown in Figure 1a. The SIMP method adopts a power function to determine the properties of a pixel, as follows for *M* materials:

(1) $$C^{\left(1,\cdots ,M\right)}=\sum_{i=1}^{M}\left\{C^{\left(M-i+1\right)}[1-{\left(\rho^{i}-\rho^{i}\delta_{iM}\right)}^{p}]\overset{i-1}{\underset{j=1}{\Pi }}{\left(\rho^{j}\right)}^{p}\right\},\delta_{iM}=\{\begin{array}{c} 1,i=M\\ 0,i\neq M \end{array},$$

where $C^{\left(1,\cdots ,M\right)}$ is the interpolated material property of the *M* materials, ***C****^i^* is the original material property of the *i*’th material, $\rho^{i}$ is the artificial density, *p* is the penalty factor, and *δ_iM_* is the Kronecker delta.

However, in the element stacking method, each pixel corresponds to multiple finite elements, and then chooses a finite element that satisfies different governing equations or has different material properties with different boundary conditions. In other words, more than one element is juxtaposed on the same pixel in the element stacking method, as shown in Figure 1b. If only the material selection is considered, this method is basically the same as the conventional multi-material topology optimization method. In order to be able to select only one finite element from multiple finite elements, a finite element selection strategy needs to be adopted and its expression is as follows:

(2) $$C^{e}=\sum_{i=1}^{M}\left\{{\left(\rho_{i}^{e}\right)}^{p}\overset{M}{\underset{j=1}{\Pi }}[1-{\left(\rho_{j\neq i}^{e}\right)}^{q}]\right\}C_{i},$$

where ***C****^e^* is the material property of the *e*’th element that is interpolated by *M* materials, $\rho_{i}^{e}$ ($0<\rho_{i}^{e}\leq 1$) is the design variable representing the selection of the *i*’th material at the *e*’th finite element; *p* and *q* are the penalty exponents and ***C****_i_* denotes the actual material property of the *i*’th material. For example, if two materials with Young’s modulus *E*_1_ and *E*_2_ are considered, the Young’s modulus of the *e*’th element can be expressed as follows:

(3) $$E^{e}=E_{1}{\left(\rho_{1}^{e}\right)}^{p}[1-{\left(\rho_{2}^{e}\right)}^{q}]+E_{2}{\left(\rho_{2}^{e}\right)}^{p}[1-{\left(\rho_{1}^{e}\right)}^{q}],$$

In order to obtain the element stiffness matrix ***k****^e^* of the *e*’th pixel with *M* stacked finite elements, the interpolation formulation of Equation (2) is slightly modified, as follows:

(4) $$k^{e}(\rho^{e})=\overset{M}{\underset{i=1}{A}}k_{i}^{e}(\rho^{e})=\overset{M}{\underset{i=1}{A}}\phi_{i}^{e}(\rho^{e}){\tilde{k}}_{i}^{e}=\overset{M}{\underset{i=1}{A}}\left[{\left(\rho_{i}^{e}\right)}^{p}\prod_{j=1,j\neq i}^{M}[1-{\left(\rho_{j}^{e}\right)}^{q}]\right]{\tilde{k}}_{i}^{e},$$

where vector $\rho^{e}={\left[\rho_{1}^{e},\rho_{2}^{e},\cdots \rho_{M}^{e}\right]}^{T}$ is the design variables, $\phi_{i}^{e}$ denotes the participation factor of the *i*th element stiffness to the interpolated stiffness of the *e*th pixel, and the stiffness matrix $k_{i}^{e}(\rho^{e})$ depends on the set of design variables $\rho^{e}$. The stiffness matrix ${\tilde{k}}_{i}^{e}$ ($\rho_{i}^{e}=1$, other $\rho_{j,i\neq j}^{e}=0$) is a design variable-independent matrix. The symbol $A_{i=1}^{M}$ denotes the pixel-level assembly operator of the element stiffness matrices $k_{i}^{e}$.

## 3. Problem Formulation for Multi-Material Flexure Hinge Design

**Figure 2:**
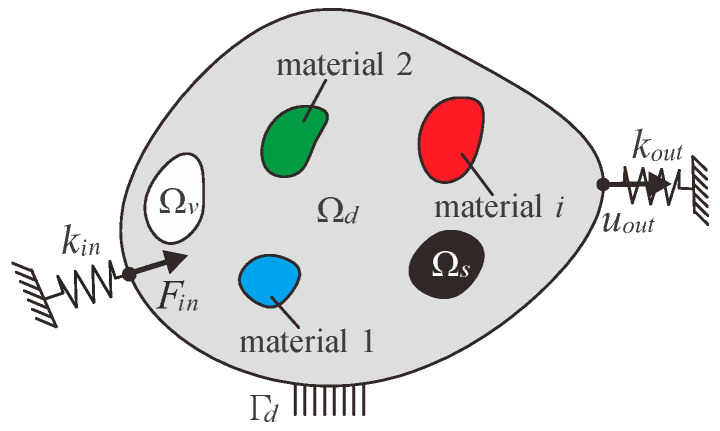
General formulation for multi-material topology optimization of compliant mechanisms.

A general problem for the topology optimization of multi-material compliant mechanisms is illustrated in Figure 2. A general linear-elastic body occupying a two-dimensional region and fixed at the boundary Γ*_d_* is considered. The elastic body can be divided into a prescribed solid domain Ω*_s_*, a prescribed void domain Ω*_v_*, and a design domain Ω*_d_*. The design domain can set *i* different materials, according to the requirements. The input port is subjected to a given force *F_in_* and an artificial spring *k_in_* is attached here which is used to simulate the interaction between the actuator and the compliant mechanism. The output port exhibits the output displacement *u_out_* due to input force *F_in_* and an artificial spring *k_out_* is also attached here which is used to simulate the interaction between the workpiece and the compliant mechanism.

In order to achieve the functional requirements of the compliant mechanism, the output displacement is generally maximized. The general optimization model for the multi-material topology optimization of compliant mechanisms using the element stacking method can be stated as follows:

(5) $$\begin{array}{ll} \min: & -u_{out}\\ s.t.: & F=K(\rho )u=\overset{N}{\underset{e=1}{A}}\left[k^{e}(\rho^{e})\right]u\\ & V_{i}=\sum_{e=1}^{N}\rho_{i}^{e}v^{e}/V_{tol}\leq V_{i}^{*},\;i=1,2,\cdots ,M\\ & \rho =\left[\rho^{1},\rho^{2},\cdots ,\rho^{N}\right],\;\rho^{e}=\left[\rho_{1}^{e},\rho_{2}^{e},\cdots ,\rho_{M}^{e}\right]\\ & 0.001=\rho_{\min}\leq \rho_{i}^{e}\leq 1,\;i=1,2,\cdots ,M,\;e=1,2,\cdots ,N. \end{array},$$

where ***K***(***ρ***) is the global stiffness matrix, ***u*** is the displacement vector due to force vector ***F***, *N* is the total number of discretizing elements, *v^e^* is the volume of the *e*th pixel, *V_tol_* is the total volume of the design domain, *V_i_* is the fraction of the volume of the *i*th material to the total volume, and V_i_^*^ is the upper limit of the volume fraction of the *i*th material.

### 3.1. Topology Optimization Model

**Figure 3:**
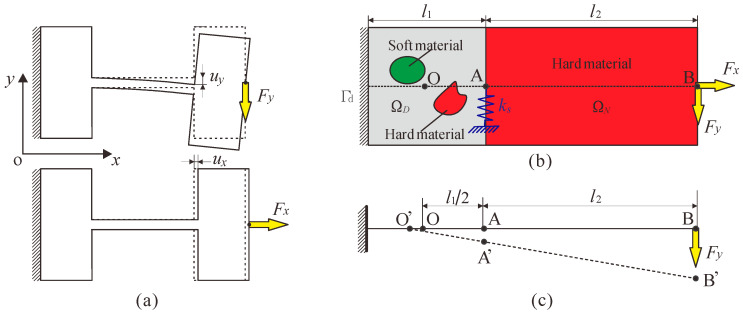
General formulation for multi-material topology optimization of flexure hinge. (**a**) A flexure hinge with boundary conditions and loads, (**b**) design domain, (**c**) schematic diagram of rotation center constraint.

A flexure hinge with boundary conditions and loads is shown in Figure 3a, in which the left end of the hinge is fixed and the other end is free. Since flexure hinges are usually used in compliant mechanisms that achieve precision and small displacement, it is modelled as a small deformation fixed-free Euler–Bernoulli beam. When the flexure hinge is subjected to a force *F_y_*, it will produce a displacement *u_y_* in the vertical direction, and when the flexure hinge is subjected to a force *F_x_*, it will produce a displacement *u_x_* in the horizontal direction. To make the flexure hinge easy to rotate, its flexibility in the y-direction should be as large as possible. However, the flexure hinge obtains rotational motion through its own deformations. In engineering applications, the flexure hinge will also be subjected to axial force, so the parasitic motion is inevitable (that is, the deformation will also occur in the x-direction). In order to make the flexure hinge closer to the rigid joint, the flexibility of flexure hinge in the y-direction should be as large as possible and the flexibility in the x-direction should be as small as possible.

Figure 3b shows the schematic diagram of the topology optimization model of a multi-material flexure hinge, where Ω*_D_* is the design domain, including soft and hard materials, and Ω*_N_* is the rigid link whose material is only one hard material and is a prescribed non-design domain. *Γ_d_* is the Dirichlet boundary condition with prescribed displacements, and *l*_1_ and *l*_2_ are the lengths of the design domain and the non-design domain, respectively. Point O is the geometric center of the design domain; points A and B are the midpoint of the right end of the design domain and the midpoint of the right end of the non-design domain, respectively. In the topology optimization of a multi-material flexure hinge, the left end of the design domain is fixed and then forces *F_x_* and *F_y_* are applied at point B. Simultaneously, an artificial spring *k_s_* is added to the y-direction of point A to turn the topology optimization problem into a well-posed design, thereby avoiding the numerical instabilities.

In topology optimization, the flexibility of flexure hinges is quantitatively represented by compliance *C* = ***F**^T^**u***. According to the aforementioned, in order to obtain an ideal flexure hinge, the compliance in the y-direction *C_y_* of the design domain needs to be maximized, while the compliance in the x-direction *C_x_* needs to be minimized. Therefore, the objective function of the topology optimization problem can be written as

(6) $$\min:f=-C_{y}+C_{x}=-F_{y}^{T}u_{y}+F_{x}^{T}u_{x},$$

where ***F_x_*** and ***F_y_*** are the force vectors, which are zero in all degrees of freedom except for the input degree of freedom, and ***u_x_*** and ***u_y_*** are the displacement vectors produced by forces *F_x_* and *F_y_*, respectively.

The traditional rigid joint can rotate around a fixed rotational center, but the rotational center of the flexure hinge will be offset due to its elastic deformation. Thus, to ensure the rotational precision of the flexure hinge, it is necessary to add a constraint condition. In this work, the geometric center O of the design domain is set as the rotational center of the hinge. When the flexure hinge is acted on by a force *F_y_*, it will produce a slight rotation. Then the intersection point O’ of the extension line of the line segment AB and the axis should be as close to the point O as possible, so that the precision of the flexure hinge will be higher. According to the geometric relationship and the small deformation assumption,

(7) $$\frac{\bar{AA^{\prime }}}{\bar{BB^{\prime }}}\approx \frac{\bar{OA}}{\bar{OB}}\to \frac{u_{y,F_{y}}^{A}}{u_{y,F_{y}}^{B}}\approx \frac{\frac{1}{2}l_{1}}{\frac{1}{2}l_{1}+l_{2}},$$

Therefore, the rotational center constraint of the flexure hinge can be expressed as follows

(8) $$\Lambda ={\left(\frac{u_{y,F_{y}}^{A}}{u_{y,F_{y}}^{B}}-\frac{l_{1}}{l_{1}+2l_{2}}\right)}^{2}\leq \Lambda^{*},$$

where $u_{y,F_{y}}^{A}$ is the displacement of point A in the y-direction due to force *F_y_*, $u_{y,F_{y}}^{B}$ is the displacement of point B in the y-direction due to force *F_y_*, and Λ^*^ is a given small positive constant and is set to 0.01 in this work.

The topology optimization model for the multi-material flexure hinge can now be formulated as follows

(9) $$\begin{array}{ll} \underset{\rho }{\min}: & f(\rho )=-C_{y}+C_{x}\\ s.t.: & K_{x}(\rho )u_{x}=F_{x}\\ & K_{y}(\rho )u_{y}=F_{y}\\ & \Lambda \leq \Lambda^{*}\\ & V_{i}=\sum_{e=1}^{N}\rho_{i}^{e}v^{e}/V_{tol}\leq V_{i}^{*},\;i=1,2,\cdots ,M\\ & \rho =\left[\rho^{1},\rho^{2},\cdots ,\rho^{N}\right],\;\rho^{e}=\left[\rho_{1}^{e},\rho_{2}^{e},\cdots ,\rho_{M}^{e}\right]\\ & 0.001=\rho_{\min}\leq \rho_{i}^{e}\leq 1,\;i=1,2,\cdots ,M,\;e=1,2,\cdots ,N \end{array}$$

where ***K_x_***(***ρ***) and ***K_y_***(***ρ***) are the global stiffness matrices when loads *F_x_* and *F_y_* are applied, respectively, which can be written in the form

(10) $$\begin{array}{l} K_{x}(\rho )=\overset{N}{\underset{e=1}{A}}\left[k^{e}(\rho^{e})\right]\\ K_{y}(\rho )=\overset{N}{\underset{e=1}{A}}\left[k^{e}(\rho^{e})\right]+K_{s} \end{array}$$

where ***K****_s_* is the stiffness matrix that contains the artificial spring *k_s_*.

### 3.2. Sensitivity Analysis

In order to solve the topology optimization problem of a multi-material flexure hinge with multiple constraints, the method of moving asymptotes (MMA) is employed, which is a gradient-based optimization algorithm. Therefore, the gradient information of the objective function and all the constraint functions need to be derived. The sensitivity of the objective function *f*(***ρ***) with respect to the design variable $\rho_{i}^{e}$ can be written as

(11) $$\frac{\partial f}{\partial \rho_{k}^{e}}={\left(u_{y}^{e}\right)}^{T}\frac{\partial k^{e}(\rho^{e})}{\partial \rho_{k}^{e}}u_{y}^{e}-{\left(u_{x}^{e}\right)}^{T}\frac{\partial k^{e}(\rho^{e})}{\partial \rho_{k}^{e}}u_{x}^{e},$$

where $u_{x}^{e}$ and $u_{y}^{e}$ are the nodal displacement vectors of the *e*th pixel due to forces *F_x_* and *F_y_*, respectively. The partial derivative $\partial k^{e}/\partial \rho_{k}^{e}$ can be derived from Equation (4) as

(12) $$\begin{array}{ll} \frac{\partial k^{e}(\rho^{e})}{\partial \rho_{k}^{e}} & =p{\left(\rho_{k}^{e}\right)}^{p-1}\prod_{j=1,j\neq k}^{M}[1-{\left(\rho_{j}^{e}\right)}^{q}]{\tilde{k}}_{k}^{e}-q{\left(\rho_{k}^{e}\right)}^{q-1}\overset{M}{\underset{\begin{array}{l} i=1\\ i\neq k \end{array}}{A}}\left[{\left(\rho_{i}^{e}\right)}^{p}\prod_{\begin{array}{c} j=1\\ j\neq i,j\neq k \end{array}}^{M}[1-{\left(\rho_{j}^{e}\right)}^{q}]\right]{\tilde{k}}_{i}^{e}\\ & =\frac{p}{\rho_{k}^{e}}\phi_{k}^{e}(\rho^{e}){\tilde{k}}_{k}^{e}-\frac{q{\left(\rho_{k}^{e}\right)}^{q-1}}{1-{\left(\rho_{k}^{e}\right)}^{q}}\overset{M}{\underset{\begin{array}{l} i=1\\ i\neq k \end{array}}{A}}\phi_{i}^{e}(\rho^{e}){\tilde{k}}_{i}^{e} \end{array}$$

Substituting Equation (12) into Equation (11), the sensitivity of the objective function is obtained as follows

(13) $$\begin{array}{ll} \frac{\partial f}{\partial \rho_{k}^{e}}= & \frac{p}{\rho_{k}^{e}}{\left(u_{y,k}^{e}\right)}^{T}{\tilde{k}}_{k}^{e}u_{y,k}^{e}-\frac{q{\left(\rho_{k}^{e}\right)}^{q-1}}{1-{\left(\rho_{k}^{e}\right)}^{q}}\overset{M}{\underset{\begin{array}{l} i=1\\ i\neq k \end{array}}{A}}{\left(u_{y,i}^{e}\right)}^{T}{\tilde{k}}_{i}^{e}u_{y,i}^{e}\\ & -\frac{p}{\rho_{k}^{e}}{\left(u_{x,k}^{e}\right)}^{T}{\tilde{k}}_{k}^{e}u_{x,k}^{e}+\frac{q{\left(\rho_{k}^{e}\right)}^{q-1}}{1-{\left(\rho_{k}^{e}\right)}^{q}}\overset{M}{\underset{\begin{array}{l} i=1\\ i\neq k \end{array}}{A}}{\left(u_{x,i}^{e}\right)}^{T}{\tilde{k}}_{i}^{e}u_{x,i}^{e} \end{array}$$

where $u_{x,k}^{e}$ and $u_{y,k}^{e}$ are the subset vectors for ${\tilde{k}}_{k}^{e}$ from $u_{x}^{e}$ and $u_{y}^{e}$, respectively.

The gradient of the rotational center constraint Λ relative to the design variable is given as

(14) $$\frac{\partial \Lambda }{\partial \rho_{k}^{e}}=2\left(\frac{u_{y,F_{y}}^{A}}{u_{y,F_{y}}^{B}}-\frac{l_{1}}{l_{1}+2l_{2}}\right)\frac{\frac{\partial u_{y,F_{y}}^{A}}{\partial \rho_{k}^{e}}u_{y,F_{y}}^{B}-\frac{\partial u_{y,F_{y}}^{B}}{\partial \rho_{k}^{e}}u_{y,F_{y}}^{A}}{{\left(u_{y,F_{y}}^{B}\right)}^{2}},$$

To solve the partial derivatives of displacement with respect to the design variable, the displacement $u_{y,F_{y}}^{A}$ can be written as $u_{y,F_{y}}^{A}=\Psi_{A}^{T}u_{y}$, where $\Psi_{A}^{T}$ is an adjoint force vector with unit force in the degree of freedom in the y-direction of point A. The term $\partial u_{y,F_{y}}^{A}/\partial \rho_{k}^{e}$ is derived as

(15) $$\frac{\partial u_{y,F_{y}}^{A}}{\partial \rho_{k}^{e}}=\Psi_{A}^{T}\frac{\partial u_{y}}{\partial \rho_{k}^{e}}=\Psi_{A}^{T}K_{y}^{-1}\left(\frac{\partial F_{y}}{\partial \rho_{k}^{e}}-\frac{\partial K_{y}}{\partial \rho_{k}^{e}}u_{y}\right),$$

where $\Psi_{A}^{T}$ can be replaced by the adjoint matrix equation to simplify the calculation as

(16) $$K_{y}{\bar{u}}_{y}^{A}=\Psi_{A},$$

where ${\bar{u}}_{y}^{A}$ is the adjoint displacement vector.

Assuming the design variable is independent of the loads, then it has

(17) $$\frac{\partial F_{y}}{\partial \rho_{k}^{e}}=0,$$

Substituting Equations (10), (12), (16), and (17) into Equation (15) gives the gradient as

(18) $$\frac{\partial u_{y,F_{y}}^{A}}{\partial \rho_{k}^{e}}=-\frac{p}{\rho_{k}^{e}}{\left({\bar{u}}_{y,k}^{A,e}\right)}^{T}{\tilde{k}}_{k}^{e}u_{y,k}^{e}+\frac{q{\left(\rho_{k}^{e}\right)}^{q-1}}{1-{\left(\rho_{k}^{e}\right)}^{q}}\overset{M}{\underset{\begin{array}{l} i=1\\ i\neq k \end{array}}{A}}{\left({\bar{u}}_{y,i}^{A,e}\right)}^{T}{\tilde{k}}_{i}^{e}u_{y,i}^{e},$$

where ${\bar{u}}_{y,k}^{A,e}$ is the subset vector from ${\bar{u}}_{y}^{A}$.

Similarly, the term $\partial u_{y,F_{y}}^{B}/\partial \rho_{k}^{e}$ is derived as

(19) $$\frac{\partial u_{y,F_{y}}^{B}}{\partial \rho_{k}^{e}}=-\frac{p}{\rho_{k}^{e}}{\left({\bar{u}}_{y,k}^{B,e}\right)}^{T}{\tilde{k}}_{k}^{e}u_{y,k}^{e}+\frac{q{\left(\rho_{k}^{e}\right)}^{q-1}}{1-{\left(\rho_{k}^{e}\right)}^{q}}\overset{M}{\underset{\begin{array}{l} i=1\\ i\neq k \end{array}}{A}}{\left({\bar{u}}_{y,i}^{B,e}\right)}^{T}{\tilde{k}}_{i}^{e}u_{y,i}^{e},$$

where ${\bar{u}}_{y,k}^{B,e}$ is the subset vector from the adjoint displacement vector ${\bar{u}}_{y}^{B}$ due to the adjoint force vector $\Psi_{B}^{T}$ with unit force in the degree of freedom in the y-direction of point B.

Substituting Equations (18) and (19) into Equation (14), the gradient of the rotational center constraint Λ can be solved.

## 4. Numerical Implementation

In this section, several design examples of multi-material flexure hinges are considered. The design domain that is used for the examples is shown in Figure 3b and is set to a square. In all examples, the design domain contains two materials: Material 1 is a hard material with Young’s modulus *E*_1_ and Poisson’s ratio *μ*_1_, which is shown in red in the topology optimization result; Material 2 is a soft material with Young’s modulus *E*_2_ and Poisson’s ratio *μ*_2_, which is shown in green in the topology optimization result. The non-design domain contains only Material 1 (hard material). Note that two finite elements with Materials 1 and 2 are juxtaposed on every pixel when the element stacking method is adopted. That is, two layers of elements are placed on the design domain and one layer of elements is placed on the non-design domain. The design domain and non-design domain are discretized into 100 × 100 and 300 × 100 four-node square elements with a thickness of 10 mm. The lengths of the design and non-design domains are set to *l*_1_ = 10 mm and *l*_2_ = 30 mm, respectively. In the design domain, the ratio of the sum of the two material volumes to the total volume is 30%. The forces *F_x_* and *F_y_* are set to be 100 N and 10 N, respectively.

First, a flexure hinge is designed based on the multi-material topology optimization model and compared with a single-material flexure hinge. The properties of these two materials are *E*_1_ = 71 GPa, *μ*_1_ = 0.3, *E*_2_ = 1 GPa, *μ*_2_ = 0.3, and the volume ratios are *V*_1_^*^ = *V*_2_^*^ = 0.15. The artificial spring stiffness *k_s_* = 800 N/m.

**Figure 4:**
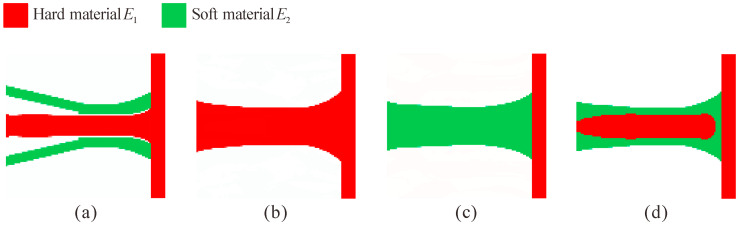
Topology optimization results for multi-material flexure hinge (**a**) *E*_1_ = 71 GPa, *V*_1_^*^ = 0.15, *E*_2_ = 1 GPa, *V*_2_^*^ = 0.15, and single-material flexure hinges; (**b**) *E*_1_ = 71 GPa, *V*_1_^*^ = 0.3, *E*_2_ = 1 GPa, *V*_2_^*^ = 0; (**c**) *E*_1_ = 71 GPa, *V*_1_^*^ = 0, *E*_2_ = 1 GPa, *V*_2_^*^ = 0.3; (**d**) *E*_1_ = 71 GPa, *V*_1_^*^ = 0.15, *E*_2_ = 71 GPa, *V*_2_^*^ = 0.15.

Figure 4a shows the topology optimization result for the multi-material flexure hinge (the entire design domain Ω*_D_* and part of the non-design domain Ω*_N_* are displayed for all examples). The topology optimization results for the single-material flexure hinges obtained from different setups are shown in Figure 4b–d, respectively. Figure 4b shows the flexure hinge with only hard materials obtained by setting the volume fraction of soft material to zero; Figure 4c shows the flexure hinge with only soft materials obtained by setting the volume fraction of hard material to zero; and Figure 4d shows the single-material flexure hinge obtained by setting the Young’s modulus of both materials as *E*_2_ = *E*_1_ = 71 GPa. As shown, the configurations of the three single-material flexure hinges are the same, and they are very similar to the corner-filleted flexure hinge. However, the configuration of the multi-material flexure hinge is quite different from that of the single-material flexure hinge, where the hard materials are distributed in the middle of the hinge and the soft materials are distributed above and below the hard materials.

**Figure 5:**
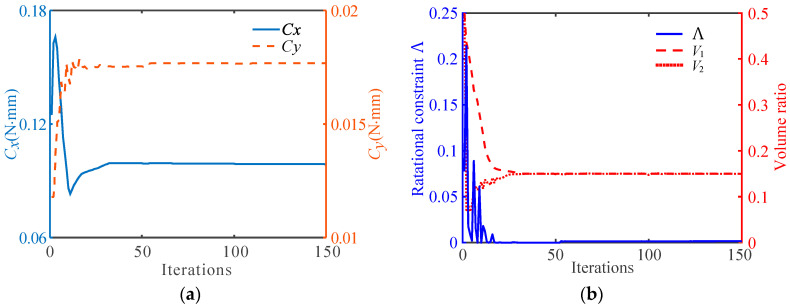
Iterative process for topology optimization of multi-material flexure hinges. (**a**) The convergence histories of the objective function, (**b**) the convergence histories of the rotational center constraint Λ and the volume fractions *V*_1_ and *V*_2_.

The convergence histories of the objective function for the obtained multi-material flexure hinge described in Figure 4a are shown in Figure 5a. It can be observed that the compliance *C_y_* in the y-direction of the flexure hinge gradually increases and the compliance *C_x_* in the x-direction of the hinge gradually decreases. Figure 5b shows the iterative process of the rotational center constraint Λ and the volume fractions *V*_1_ and *V*_2_. It can be seen that the Λ finally converges to a value that is very close to 0, and the volume constraints of both materials converge to the given value of 0.15.

Generally, the force-deformation relationship at point A (as shown in Figure 3b) is used to represent the performance of the flexure hinge. Since all deformations of the hinge are sufficiently small, the relationship of the displacements at point A and loads at point B can be represented as a linear system of equations as follows:

(20) $$\left[\begin{array}{c} \theta \\ u_{y}^{A}\\ u_{x}^{A} \end{array}\right]=\left[\begin{array}{ccc} C_{\theta ,M^{A}} & C_{u_{y}^{A},M^{A}} & 0\\ C_{\theta ,F_{y}^{A}} & C_{u_{y}^{A},F_{y}^{A}} & 0\\ 0 & 0 & C_{u_{x}^{A},F_{x}^{A}} \end{array}\right]\left[\begin{array}{c} M^{A}\\ F_{y}^{A}\\ F_{x}^{A} \end{array}\right]=\left[\begin{array}{ccc} C_{\theta ,M^{A}} & C_{u_{y}^{A},M^{A}} & 0\\ C_{\theta ,F_{y}^{A}} & C_{u_{y}^{A},F_{y}^{A}} & 0\\ 0 & 0 & C_{u_{x}^{A},F_{x}^{A}} \end{array}\right]\left[\begin{array}{c} F_{y}\left(l_{1}+2l_{2}\right)/2\\ F_{y}\left(l_{1}+2l_{2}\right)/l_{1}\\ F_{x} \end{array}\right],$$

where $M^{A}$, $F_{y}^{A}$, and $F_{x}^{A}$ are the moment, vertical force, and axial force that are applied at point A, respectively. Each element in the compliance matrix is called a compliance factor and the derivation process is detailed in reference [29].

In order to quantitatively compare the performances of multi-material and single-material flexure hinges, the rotation ratio *Rr* is defined as follows:

(21) $$R_{r}=\frac{C_{u_{y}^{A},F_{y}^{A}}}{C_{u_{x}^{A},F_{x}^{A}}}=\frac{l_{1}}{l_{1}+2l_{2}}\frac{F_{x}}{F_{y}}\frac{{\hat{u}}_{y,F_{y}}^{A}}{u_{x,F_{x}}^{A}},$$

where $u_{x,F_{x}}^{A}$ is the displacement of point A in the x-direction due to force *F_x_*, ${\hat{u}}_{y,F_{y}}^{A}$ is the displacement of point A in the y-direction due to force *F_y_* (note that the global stiffness matrix that is used to solve ${\hat{u}}_{y,F_{y}}^{A}$ does not contain the artificial spring *k_s_*). The larger *Rr*, the closer the flexure hinge is to the rigid joint.

**Figure 6:**
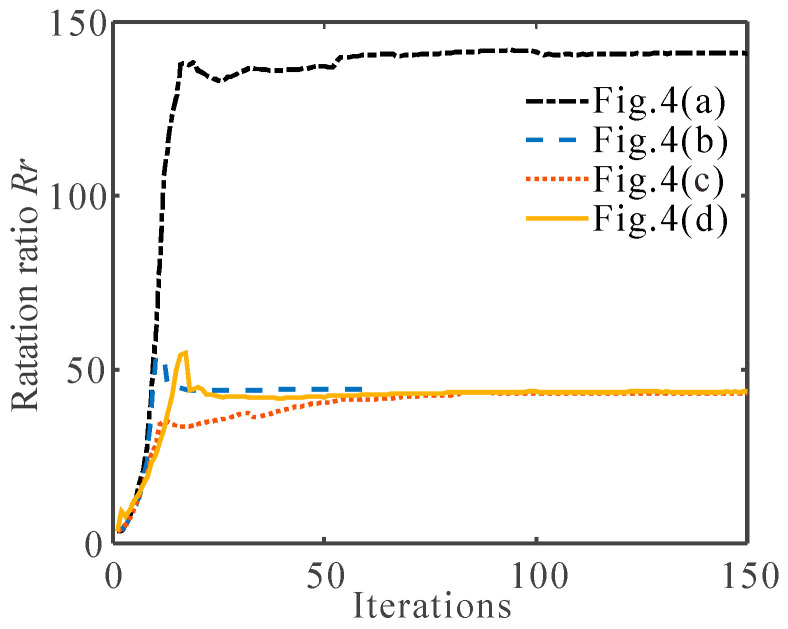
The convergence histories of the rotation ratio *R_r_* for the obtained flexure hinges described in Figure 4.

Figure 6 shows the rotation ratio for the multi-material and single-material flexure hinges. It can be seen that the rotation ratio of the multi-material flexure hinge is much larger than that of the single-material flexure hinge, and the rotation ratios of the two are 140.9 and 43.7, respectively. The numerical results show that the flexure hinge designed by using multi-material topology optimization can achieve a higher rotation ratio than the flexure hinge designed by using single-material topology optimization, making it closer to the ideal revolute joint.

### 4.1. Effect of Young’s Modulus of the Two Materials

In order to investigate the effects of Young’s modulus on the topology optimization results of a multi-material flexure hinge, several numerical examples with different Young’s modulus *E*_2_ are performed. 

**Figure 7:**
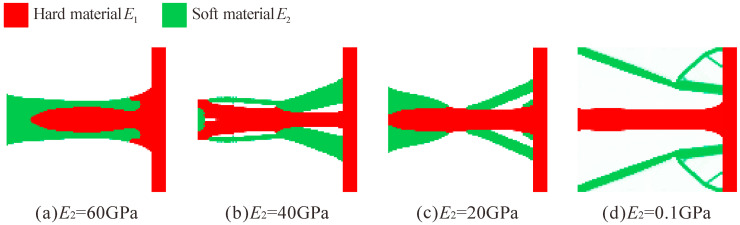
Topology optimization results of multi-material flexure hinge for different Young’s modulus combinations. (**a**) *E*_2_ = 60 GPa, (**b**) *E*_2_ = 40 GPa, (**c**) *E*_2_ = 20 GPa, (**d**) *E*_2_ = 0.1 GPa (*E*_1_ = 71 GPa for all cases).

Figure 7 shows the topology optimization results for different *E*_2_ = 60, 40, 20, and 0.1 GPa, and *E*_1_ = 71 GPa for all cases. It can be observed that the configurations of the flexure hinges that are obtained by the combination of different Young’s modulus are very different. When the Young’s modulus of the two materials is close, the configuration of the obtained multi-material flexure hinge is not much different from that of the single-material flexure hinge.

**Figure 8:**
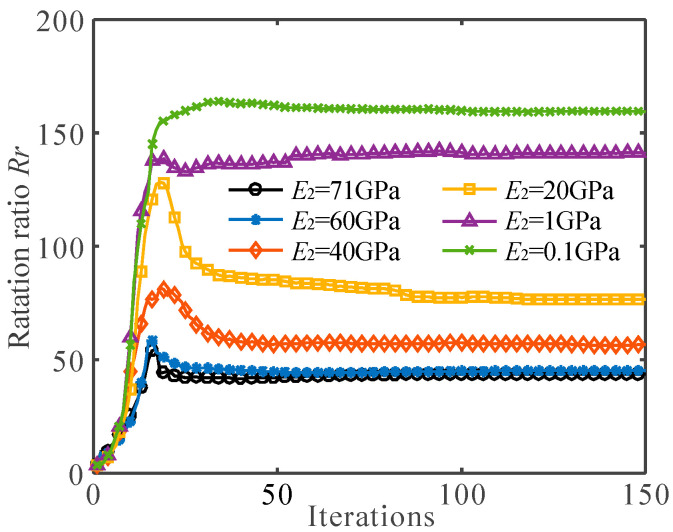
The convergence histories of the rotation ratio *R_r_* for different Young’s modulus combinations (*E*_1_ = 71 GPa).

Figure 8 shows the rotation ratio of the flexure hinges, as described in Figure 4a,d and Figure 7. When the Young’s modulus of the soft material is close to the Young’s model of the hard material (*E*_1_ = 71 GPa, *E*_2_ = 60 GPa), the rotation ratio of the multi-material flexure hinge is 45.2, which is not much different from the rotation ratio of the single-material flexure hinge. When the Young’s modulus of the soft material is very small (*E*_2_ = 0.1 GPa), the rotation ratio of the multi-material flexure hinge is as high as 159.2, which is much larger than that of the single material flexure hinge. It can also be clearly observed from Figure 8 that with the decrease in *E*_2_, the rotation ratio gradually increases. These examples indicate that the greater the difference between the Young’s modulus of the two materials, the greater the rotation ratio of the flexure hinge. When the Young’s modulus of the two materials is closer, the multi-material flexure hinge is closer to the single-material flexure hinge.

### 4.2. Effect of Volume Fraction of the Two Materials

In order to further investigate the effects of volume fraction on the topology optimization results of a multi-material flexure hinge, several numerical examples with different volume fraction are applied. In all examples, the ratio of the total volume of soft and hard materials to the design domain volume is 30%.

**Figure 9:**
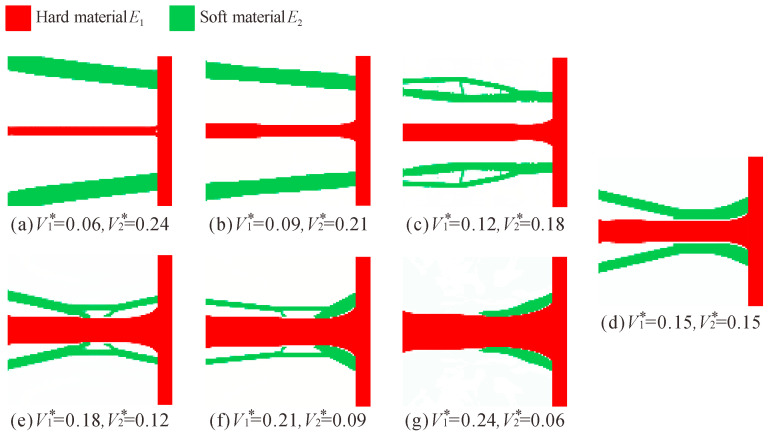
Topology optimization results of multi-material flexure hinge for different volume fraction combinations (*E*_1_ = 71 GPa, *E*_2_ = 1 GPa).

Figure 9 shows the topology optimization results for different volume fraction combinations under the conditions of Young’s modulus *E*_1_ = 71 GPa and *E*_2_ = 1 GPa. First, it can be clearly seen that the topology optimization results of the multi-material flexure hinge change with the change of the volume fraction.

In Figure 9d, the volume of the two materials is equal, and the volume of the hard material in Figure 9a–c is less than that of the soft material. From Figure 9a–c, it can be observed that as the volume of the hard material decreases (the volume of the soft material increases), the soft material gradually moves away from the hard material and tends to be trapezoidal. In Figure 9e–g, the volume of the hard material is more than that of the soft material. As the volume of hard material increases (the volume of soft material decreases), the configuration of the flexure hinge gradually moves towards the leaf-type flexure hinge, and when the volume of the soft material is very small (*V*_1_^*^ = 0.24 and *V*_2_^*^ = 0.06), its effect can be ignored. 

Although the configurations of these multi-material flexure hinges are quite different, they have one thing in common: the hard materials are distributed in the middle and the soft materials are distributed in the upper and lower sides. The possible reason is that the Young’s modulus of the soft material is much smaller than that of the hard material. 

**Figure 10:**
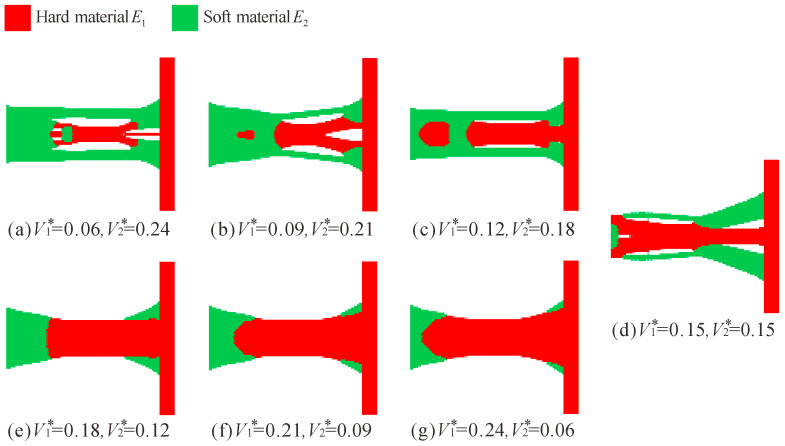
Topology optimization results of multi-material flexure hinge for different volume fraction combinations (*E*_1_ = 71 GPa, *E*_2_ = 40 GPa).

In order to analyze how the configuration of the flexure hinge changes when the Young’s modulus of the two materials is not much different, the Young’s modulus of the hard and soft materials is set to *E*_1_ = 71 GPa and *E*_2_ = 40 GPa, respectively, and the volume fractions are the same as before. The topology optimization results of the multi-material flexure hinges are shown in Figure 10. 

Similarly, as the volume fraction changes, the topology optimization results of flexure hinges also vary greatly. However, as the volume of the hard material decreases (the volume of the soft material increases), the soft material does not gradually move away from the hard material, but wraps around the hard material, as shown in Figure 10a–c. As the volume of the hard material increases, the multi-material flexure hinge likewise gravitates towards the leaf-type flexure hinge, as shown in Figure 10e–g.

**Figure 11:**
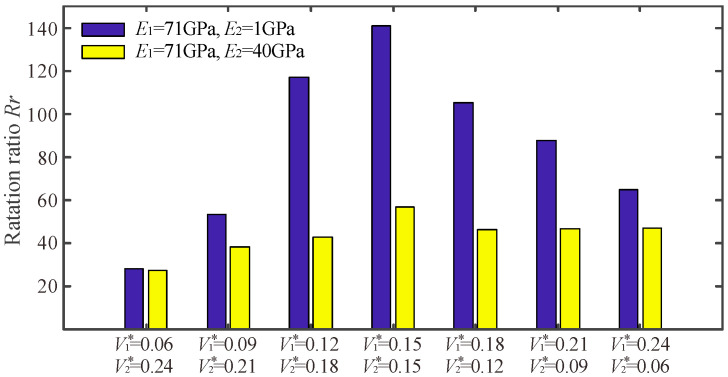
The rotation ratio *R_r_* for different volume fraction combinations.

To quantitatively analyze the effect of volume fraction on the performance of multi-material flexure hinges, the rotation ratio of the flexure hinge was calculated and displayed as a bar graph, as shown in Figure 11. It can be found that no matter the degree of difference between the Young’s modulus of the two materials, the rotation ratio of the flexure hinge reaches the maximum value when the volume of the soft and hard materials is the same. When the difference between the volumes of the soft and hard materials becomes increasingly large, the rotation ratio of the multi-material flexure hinge becomes gradually smaller.

## 5. Conclusions

In this paper, a topology optimization method for the design of multi-material flexure hinges was proposed based on the element stacking method. The topology optimization model for the multi-material flexure hinges was established based on the defined objective function and constraint functions. This was achieved by maximizing the compliance in the rotational direction of the flexure hinge while minimizing the compliance in the axial direction as the objective function. The constraint functions include the rotational center-position constraint that is used to ensure precision, and the volume fraction for various materials. The optimization problem of the multi-material flexure hinge was formulated using the element stacking method and was solved using the MMA algorithm. 

Several multi-material flexure hinges were designed to verify the effectiveness of the proposed method. It was shown that the configuration of the multi-material flexure hinge was quite different from that of the single-material flexure hinge, and its rotation ratio was larger than that of the single-material flexure hinge, indicating that a flexure hinges that is designed with a combination of soft and hard materials can achieve a better performance. The effect of the Young’s modulus of the two materials was studied. The results show that the greater the difference between the Young’s modulus of the two materials, the greater the rotation ratio of the flexure hinge. In addition, the effect of the volume fraction was investigated. It was shown that when the volumes of the two materials are the same, the rotation ratio is the largest, and when the volume difference between the two materials is larger, the rotation ratio is smaller.
